# DNA as a polyionic ionophore for barium sensor

**DOI:** 10.1186/s13065-025-01519-w

**Published:** 2025-06-19

**Authors:** M. M. Zareh, A. F. El-Farargy, A. Abd-ElSattar, Eman Rabie Abd-El-Rady, Badr Abd-El-wahaab

**Affiliations:** Department of Chemistry, Faculty of Science, Zagazig University, Zagazig, Egypt

**Keywords:** DNA-based sensor, Coated wire, Barium determination

## Abstract

The development of a highly selective and sensitive Ba^2+^ sensor is crucial because of its industrial, environmental, and biological relevance. This study introduces a novel coated wire barium-selective electrode incorporating DNA as an ecofriendly natural ionophore in a plastic membrane, utilizing dioctyl phthalate as a plasticizer. The use of DNA as an ionophore provides enhanced selectivity and sensitivity, showcasing a slope of 33.15 mV/decade across a broad concentration range (1 × 10⁻^5^ to 1 × 10⁻^2^ M). The sensor exhibited a rapid response time of 9 s, a wide pH tolerance (2.6–6.9), and good selectivity for Ba^2+^ over other cations. Characterization of the membrane using FT-IR, SEM, and EDX confirmed its structural and morphological features. Practical applicability was demonstrated by detecting Ba^2+^ in spiked samples (milk, juice, tap water and urine) with recovery rates of 96.07–98.9%. This DNA-based approach offers a promising advancement in ion-selective electrode technology, with significant implications for real-world Ba^2+^ detection.

**Graphical Abstract:**

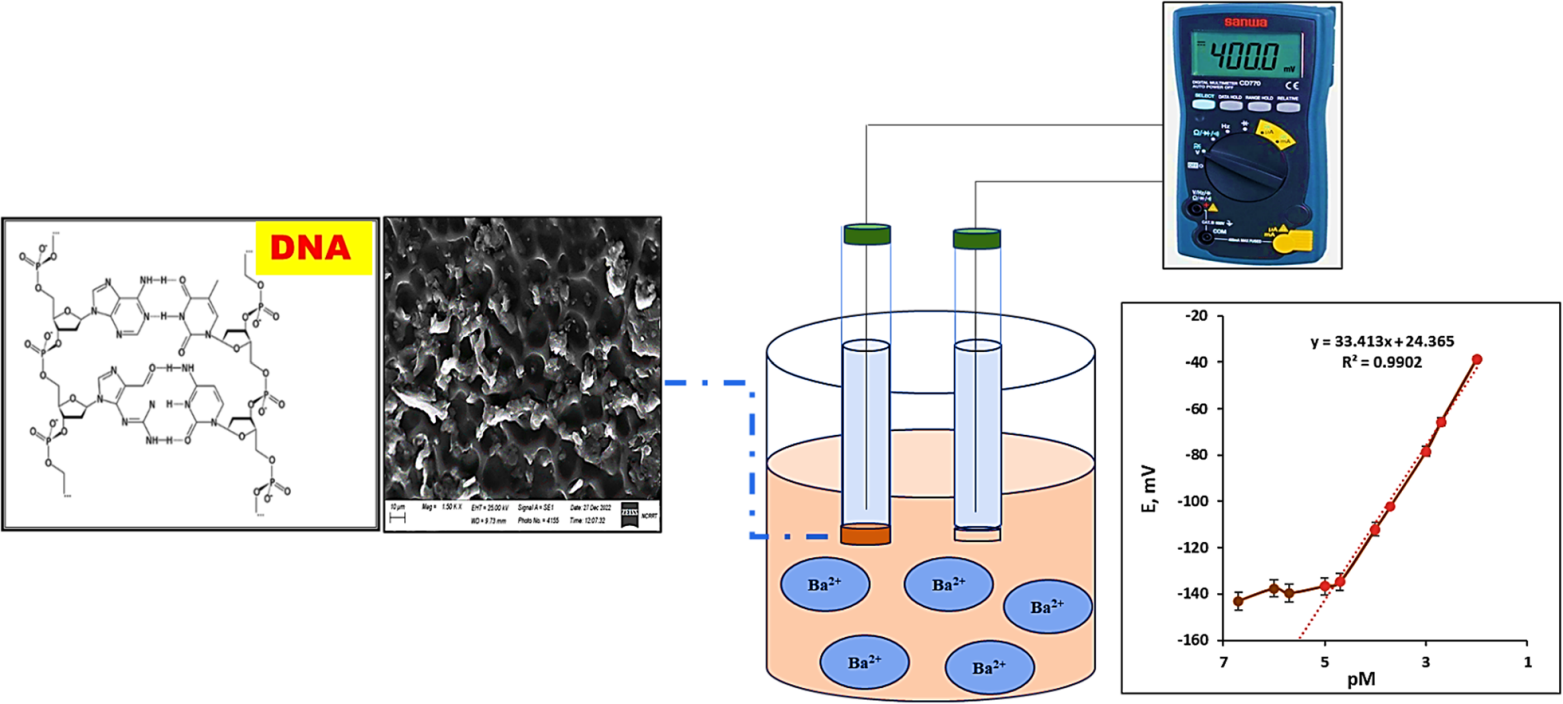

## Introduction

Selectively sensing heavy metal ions has been of great interest over decades [1–5]. Among these, barium has been present in many industrial applications, like the steel industry, gas and oil industries, the electronic industry as a dielectric layer, and the manufacture of ceramics [6, 7]. Dibello et al. applied barium in glass, dyes, bricks, and rubber manufacture. It can be used in alloys, other industries such as the paint industry, and the manufacture of lithopone. In addition, it was applied in the paint industry as a pigment in white paints. Barium is used in X-ray applications for medical functions [8]. Qiu et al. found that barium has a negative effect on protein and the enzymes of the human body because high levels of barium cause medical human problems; it has an effect on the functions of the digestive system and blood pressure and can cause respiratory failure [9]. Barium is present in food in many forms, such as nuts, fish, and some plants [10]. The previous literature recorded that the presence of barium and its compounds in water in high concentration causes toxic effects on the human body, plants, and animals [11, 12].

Various methods have been employed for the determination of barium ions, including fluorescence spectroscopy [13], flame atomic absorption spectroscopy [14], and inductively coupled plasma mass spectrometry (ICP-MS) [15, 16], each offering high sensitivity but often requiring expensive instrumentation and complex procedures. To address these limitations, electrochemical methods have emerged as efficient and straightforward alternatives for barium ion detection [17]. Among these electrochemical methods are ion-selective electrodes (ISEs), where several innovative designs have been reported for barium detection. Zamani et al. [18] developed a PVC-membrane electrode using dimethyl 1-acetyl-8-oxo-2,8-dihydro-1H-pyrazolo[5,1-a]isoindole-2,3-dicarboxylate as the sensing material, achieving a fast response time of 10 s. Similarly, Mizani et al. [19] utilised a dibenzo-17-crown-6 derivative for an ISE, which demonstrated a response time of 18 s. Othman et al. [20] prepared an electrode based on a barium-rose Bengal complex, with a response time of 20 s and a broad pH range of 4.5–10. Furthermore, Zamani et al. [21] constructed a PVC-electrochemical sensor using a thiosemicarbazone derivative, highlighting the versatility of ISEs in selecting suitable ionophores for specific applications.

**Fig. 1:**
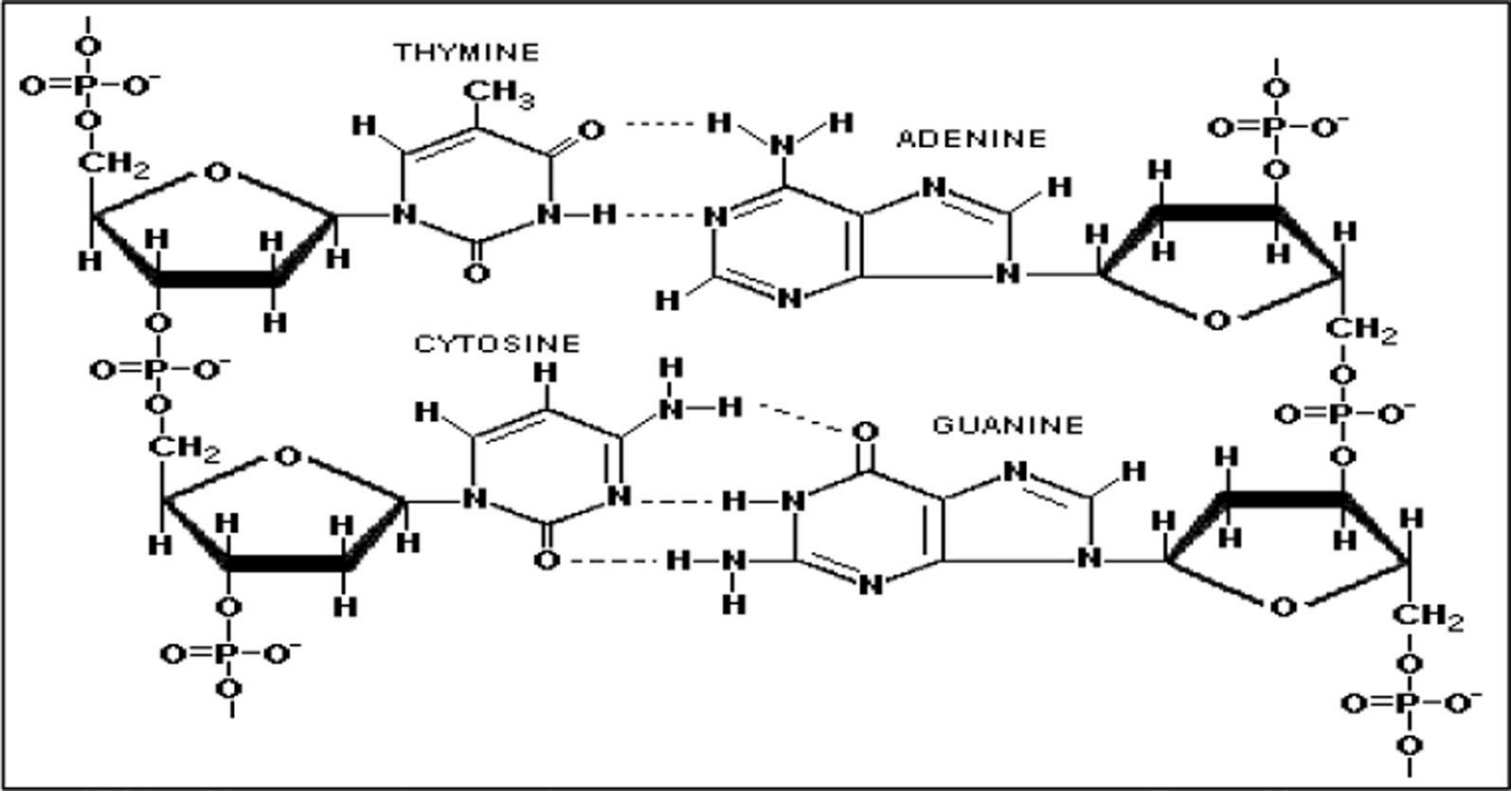
Structure formula of DNA

Compared to traditional methods, DNA-based sensors provide additional advantages, such as fast response, higher selectivity, stability, and adaptability, making them promising alternatives for precise and reliable detection of metal ions in environmental, industrial, and biological systems under various conditions [22, 23]. DNA, a molecule composed of nucleotides, consists of phosphate and sugar molecules as its primary structural components (Fig. 1). Its unique chemical structure makes it exceptionally suited for cationic binding, providing a strong foundation for its role as an ionophore. The phosphate groups in DNA can form bonds with metal ions, either directly or indirectly, through the hydrogen bonding of coordinating water molecules surrounding the metal ions [24, 25]. An electrochemical sensor based on DNAzyme was created for the extremely sensitive detection of Pb^2+^ with a detection limit of 1.74 × 10^−14^ mol·L^−1^ [26]. A novel entropy-driven multicolor DNA nanoflower was employed for the rapid and accurate detection of Pb^2+^, Cu^2+^, and Hg^2+^ in water [27]. In addition, a comparison between ISEs based on the traditional ionophores and DNA-based for barium determination was conducted in Table 4.

In this work, DNA was employed for the first time as a natural ionophore in a PVC-plastic membrane for Ba^2+^ potentiometric measurement, and its function in both sensitivity and selectivity was investigated. DNA's phosphate backbone, which offers many binding sites for metal ions, was essential to the achievement of selectivity and sensitivity toward barium ion. As a plasticiser, dioctyl phthalate improved the membrane's stability and flexibility, guaranteeing the best distribution and mobility inside the matrix. By serving as a supporting matrix, the PVC membrane guaranteed the active ingredients' homogeneous distribution and mechanical stability. By combining these materials, a strong platform for Ba^2+^ detection was produced, providing a novel substitute for conventional sensors and reducing the need for dangerous chemicals.

## Results and discussion

### Effect of membrane composition

**Fig. 2:**
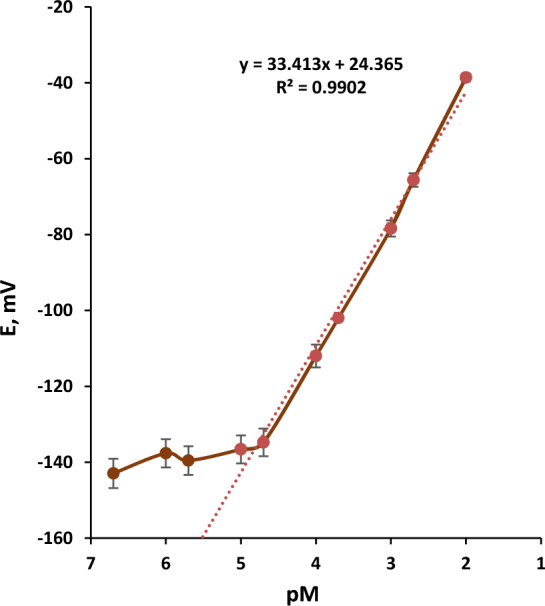
Calibration graph for a barium biosensor based on DNA with DOP as a plasticizer and containing 2 mg of DNA (I) [Average %error = 2.7]

The sensitivity and selectivity of an ion-selective electrode depend on the membrane components (ionophore, plasticizer and matrix) [28]. Different membrane compositions were tried for optimization. Six membranes were prepared by using two different plasticisers: DOP for types (I, II, and III) and DEP for types (IV, V, and VI). The slope value for sensor type-I was 33.15 mV/decade^−1^, the detection limit was 1.0 × 10^–5^ M, and the linear range was 10^–5^-10^–2^ M. This sensor-I was the best among the tested sensors as it exhibited the best performance characteristics. Other sensors (II, III, IV, V, and VI) showed a narrow linear range, which limited their use. Table 1 showed the performance of the sensor types (I-VI) for Ba^2+^ DNA-based sensors. Figure 2 showed the calibration graph of sensor type-I with the best performance characteristics.

**Table 1:** Composition and performance of Ba-DNA based sensors with membrane types- (I-VI)

Sensor No	PVC, (mg)	DNA, (mg)	DOP, (mg)	DEP, (mg)	TPB, (mg)	Slope*, mV/decade	Detection Limit*,M)	Linear Range*, (M)
I	60	2	120	–	2	33.15	1.5 × 10^–5^	10^–2^–10^–5^
II	60	4	120	–	2	13.44	1.9 × 10^–3^	10^–2^–10^−3^
III	60	6	120	–	2	3.46	1.5 × 10^–3^	10^–2^–10^–3^
IV	60	2	–	120	2	43.47	2 × 10^–4^	10^–2^–10^–4^
V	60	4	–	120	2	2.68	1.9 × 10^–3^	10^–2^–10^–3^
VI	60	6	–	120	2	13.67	1.5 × 10^–3^	10^–2^–10^–3^

^*^n = 4 times

A confidence interval (CI) was a range of estimates for an unknown parameter. A confidence interval was computed at a designated confidence level; the 95% confidence level was most common, but other levels, such as 90% or 99%, were sometimes used [29, 30]. The calculated average % errors were 2.7 for I-sensor. So, the confidence levels were 97.3% and 95.6% for the mentioned sensors.

**Fig. 3:**
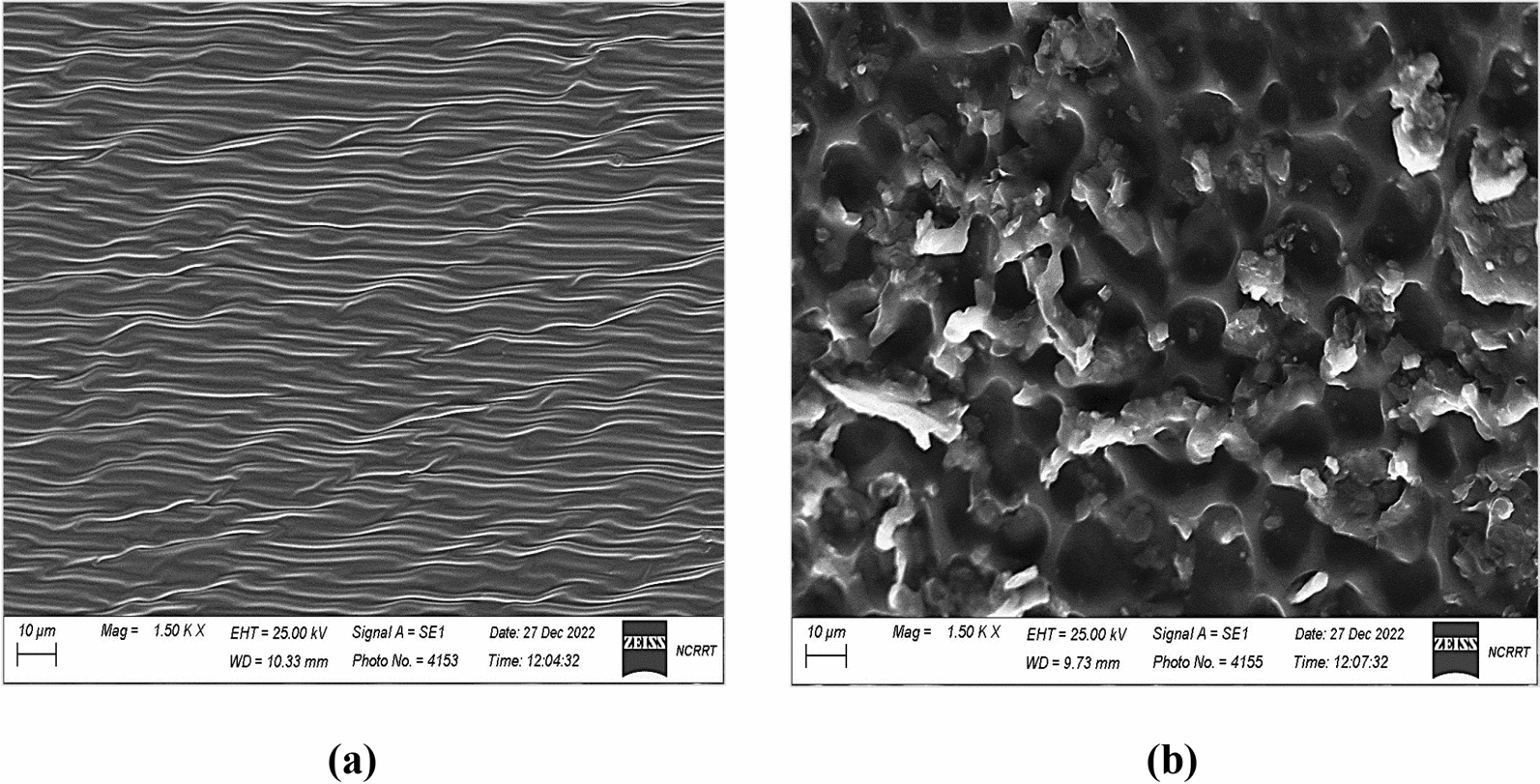
**a** SEM-photo of the blank membrane containing only (DOP + PVC), and **b** SEM- photo of the membrane type-I containing (DOP + PVC + DNA + TPB)

Reagents such as DNA and TBP changed the morphology of the membrane surface. Scanning electron microscope (SEM) and EDX showed the characterization and the changes of the surface of the membrane (Fig. 3a and b).

The SEM photos in Fig. 3a showed the PVC orientation with the polyvinylchloride arrangement like smooth and straight lines. In Fig. 3b, when DNA and TPB-K-salt were comprised into the membrane, the distribution of these compounds was observed between PVC lines, showing a surface with grooves appeared in the membrane structure. These grooves were a good surface for enhancing the adsorption of the measured ionic species. This process helped the equilibrium mechanism between the DNA and Ba^2+^ ions.

**Fig. 4:**
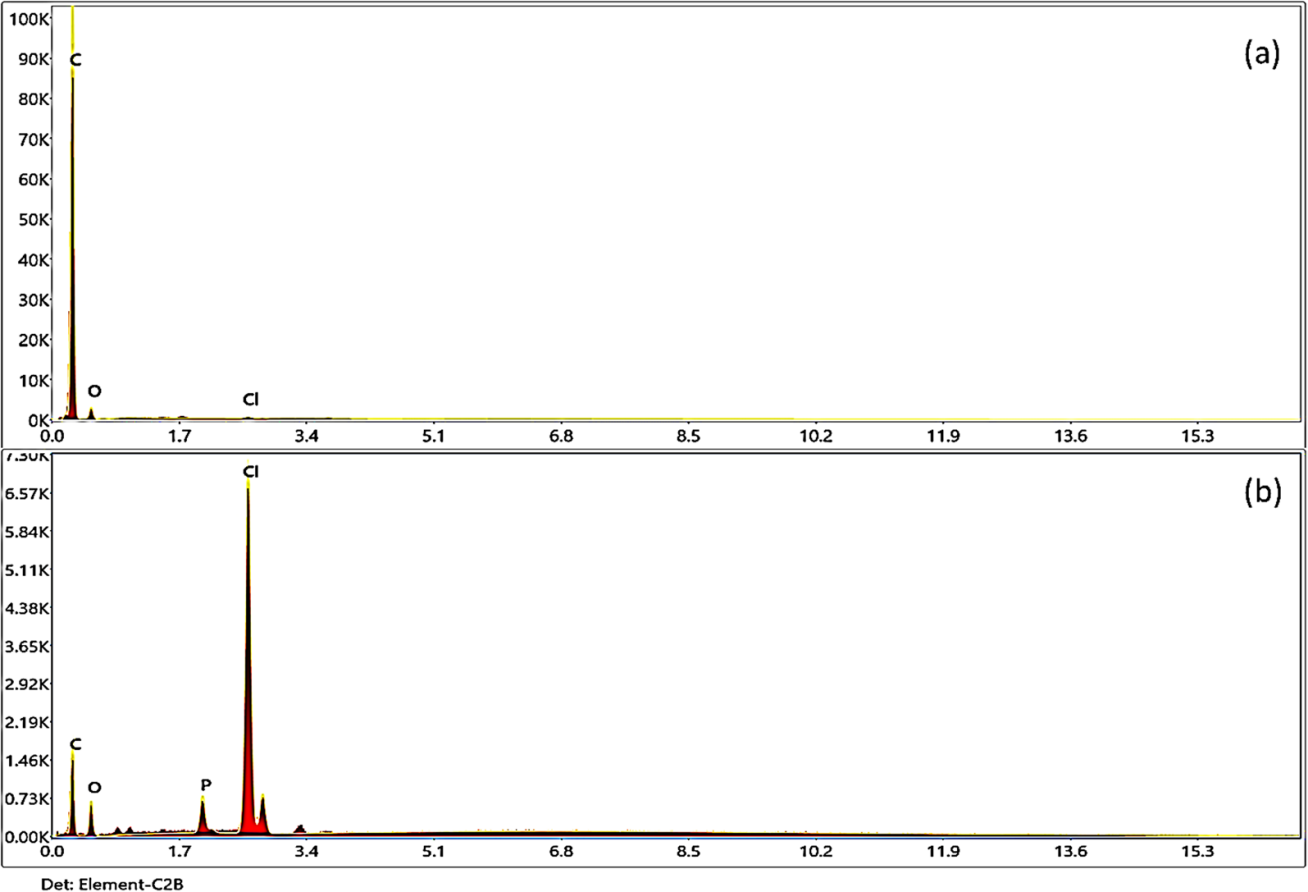
**a** EDX spectrum analysis of blank membrane containing only (DOP + PVC), and **b** EDX spectrum analysis of the membrane type-I containing (DNA + TPB)

From EDX analysis, Fig. 4a showed that the blank membrane contains 0.04% chlorine, 6.43% oxygen, and 93.53% carbon. On the other hand, Fig. 4b showed the EDX analysis for type I (with DNA and TPB-K) contains 73.11% carbon, 2.44% nitrogen, 14.57% oxygen, 0.87% phosphate, 8.73% chlorine, and 0.29% potassium. The results agreed with the morphological features that should be present for blank membranes and DNA membranes. The presence of P and N confirmed the presence of DNA in the membrane, while the presence of potassium and high Cl percent was proof of the presence of potassium tetra-(p-chlorophenyl) borate.

**Fig. 5:**
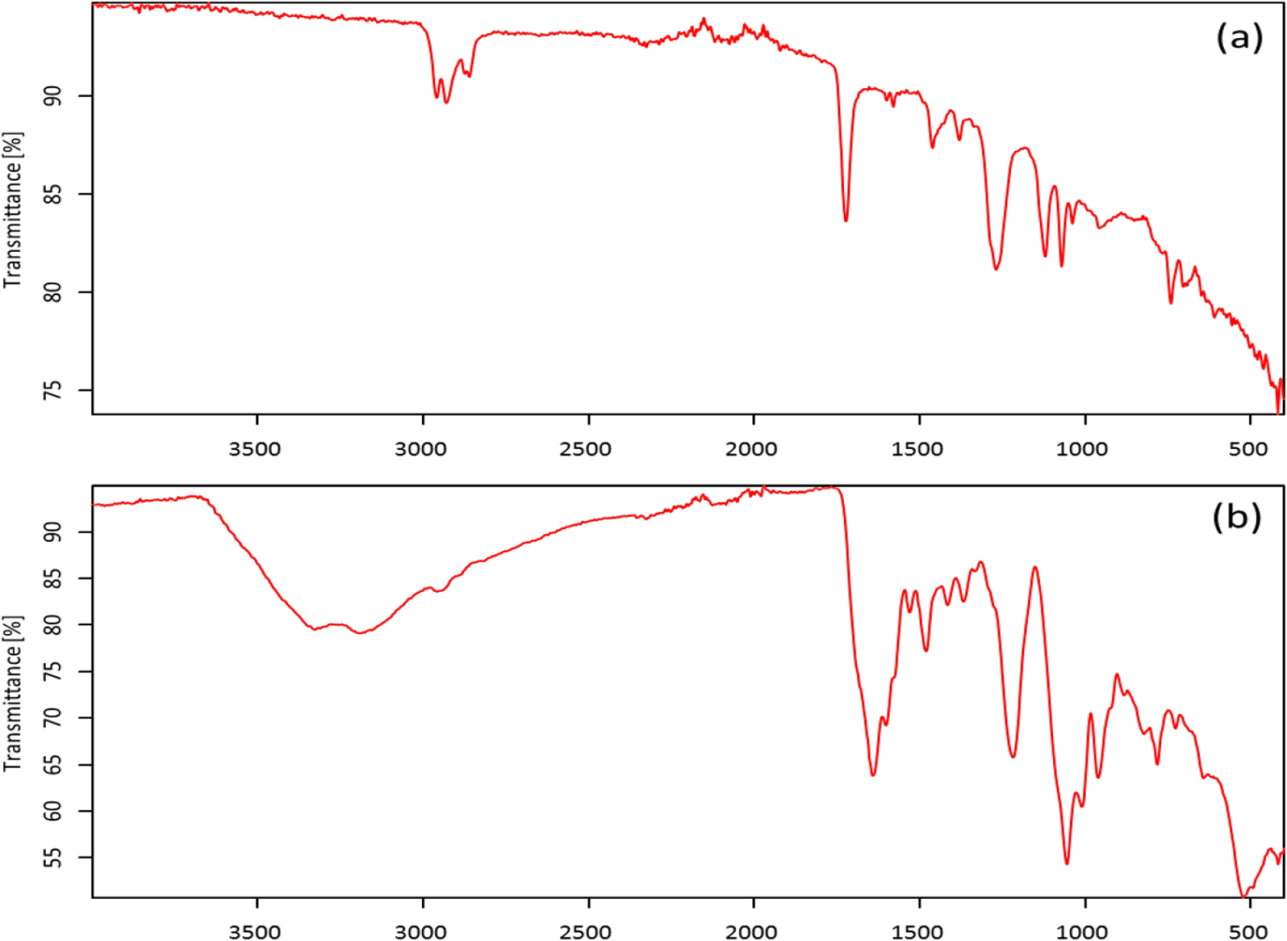
**a** FT-IR spectra of blank membrane comprising only PVC + DOP, and **b** FT-IR spectra of membrane type-I comprising DNA

FT-IR techniques exhibited stretching and binding vibration spectra of the blank membrane. Figure 5a showed the peaks for PVC at 2972 cm⁻^1^ and 2910 cm⁻^1^ for –CH_₂._ The peaks at 1400 cm⁻^1^ for the C–H bond, a peak at 1250 cm⁻^1^ for C–H near Cl, and a peak at 1000–1100 cm⁻^1^ for the C–C stretching bond of the PVC were observed. For DOP CH (1, 2, 3) stretching vibrations in the region from 2800 to 3000 cm⁻^1^, a peak at 1728 cm⁻^1^ for C=O and peaks for C–C of the aromatic ring at 1600 cm⁻^1^ and 1581 cm⁻^1^ were observed [31, 32].

The FT-IR for the membrane type-I (with DNA and TPB) was shown in Fig. 5b. The DNA peaks were observed between 1800 and 700 cm⁻^1^. The base-sugar peaks depend on glycosidic torsion angle and were found between 1500 and 1250 cm⁻^1^, while the phosphate groups showed peaks between 1250 and 1000 cm⁻^1^, and due to the phosphodiester chain coupled to vibrations of the sugar, the sugar bands were below 1000 cm⁻^1^ [33]

### Response mechanism

The sensor response depended on a non-polarized electrochemical equilibrium [34]. The phosphate groups of the DNA were good sites for the association with barium ions (K_sp_ of Ba-phosphate = 6 × 10^–39^) [35, 36]. The response mechanism of the proposed Ba-sensor was explained by two equilibrium steps. The first step was the equilibrium between Ba^2+^ ions in the solution and the membrane, where non-polarized equilibrium likely refers to the steady-state distribution of barium ions between the solution phase and the membrane phase as indicated by Eq. (1):

1 $$\left[ {{\text{Ba}}^{{{2} + }} } \right]_{{\text{s}}} \rightleftharpoons \left[ {{\text{Ba}}^{{{2} + }} } \right]_{{\text{m}}}$$

The second step was the formation of Ba^2+^–DNA into the membrane site where the equilibrium facilitates the selective binding of Ba^2^⁺ ions by the DNA phosphate groups as shown by Eq. (2):

2 $$\left[ {{\text{Ba}}^{{{2} + }} } \right]_{{\text{m}}} + {\text{ DNA}} - \left( {{\text{O}} - {\text{PO}}_{{3}} } \right)^{ - } \rightleftharpoons \left[ {{\text{DNA}} - \left( {{\text{O}} - {\text{PO}}_{{3}} } \right)^{ - } {\text{Ba}}^{{{2} + }} } \right]^{ - }_{{\text{m}}}$$

**Fig. 6:**
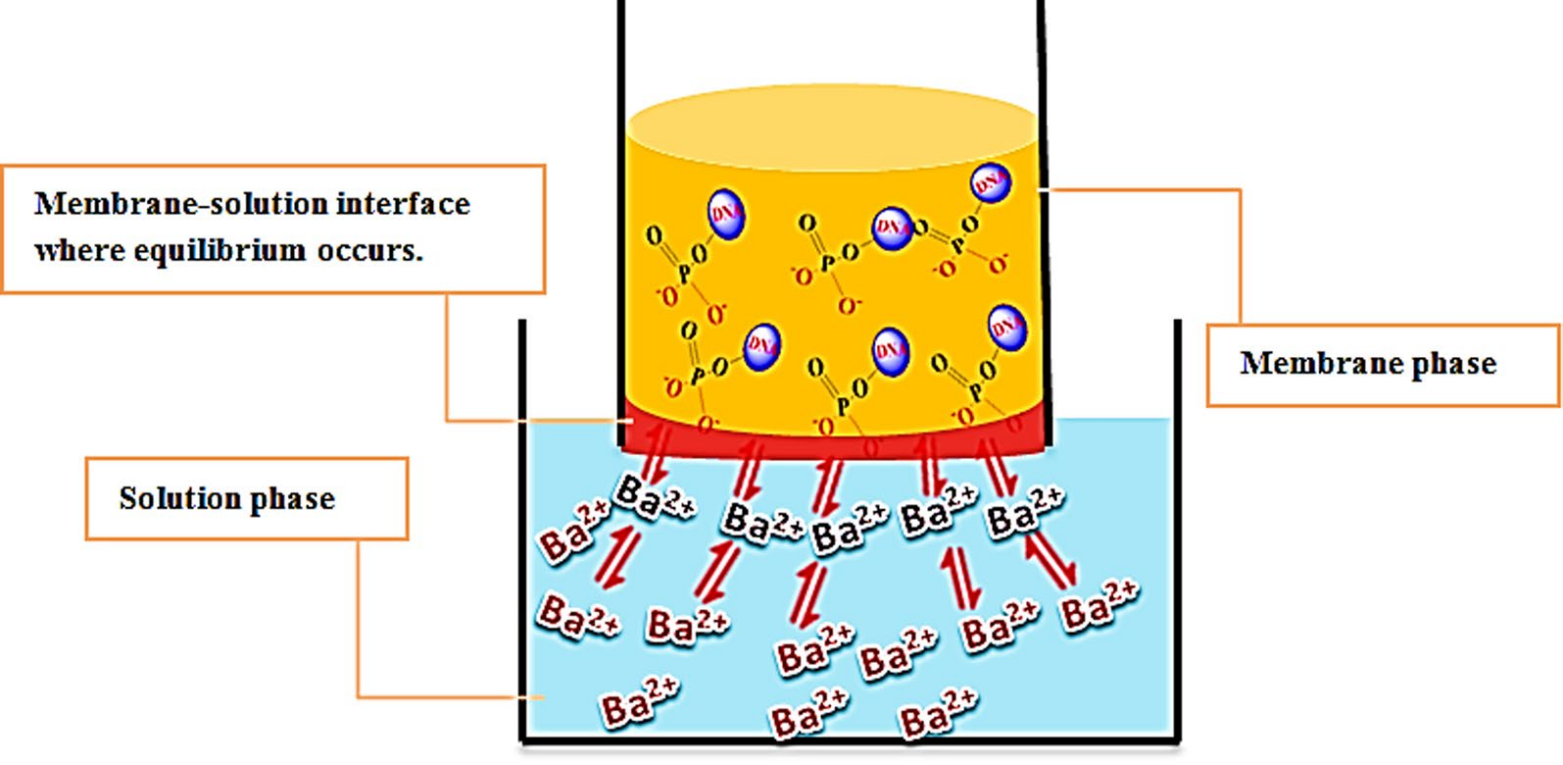
Ba^2+^-sensor response mechanism showing the two equilibrium steps

At equilibrium, the rate of Ba^2^⁺ ions binding to the DNA in the membrane matches the rate of their dissociation. This binding and dissociation equilibrium results in a potential difference across the membrane, which reflects the activity of Ba^2+^ions. The suggested response mechanism was indicated in Fig. 6.

### Effect of pH

**Fig. 7:**
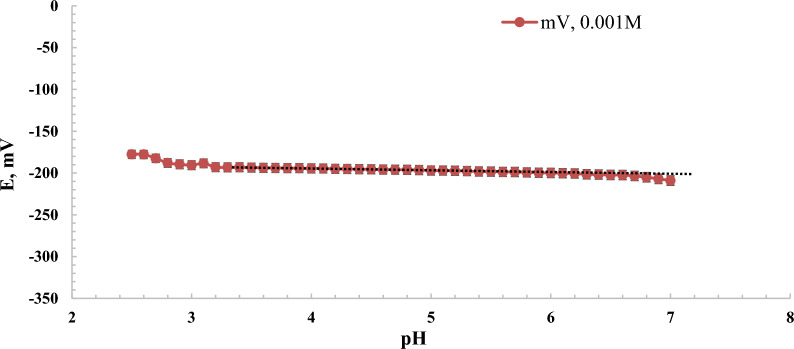
pH-effect on potential change when measuring barium solutions 10^–3^ M by using sensor type-I

One of the performance characteristics of the sensor was its working pH-range [37]. The working pH-range of sensor-I was tested using 10^–3^ M BaCl_2_ solutions, with the pH adjusted between 2 and 10 by adding drops of 0.1 M hydrochloric acid or sodium hydroxide. The potential-pH changes for sensor-I, illustrated in Fig. 7, revealed that the sensor exhibited optimal functionality in a pH range of 2.6–6.9 for the 10^–3^ M solution. This range was identified as the independent operational pH range, providing a stable environment for the interaction between Ba^2+^ ions and DNA, ensuring optimal sensor performance. Outside this range, several factors may compromise the sensor’s functionality. At highly acidic conditions (pH < 2.6), the protonation of DNA phosphate groups reduces their capacity to interact selectively with Ba^2+^ ions, thereby impairing sensor performance. In contrast, in more basic conditions (pH > 6.9), potential hydrolysis or structural changes might affect DNA stability, weakening the ionophore's ability to bind Ba^2+^. Furthermore, competing interactions with hydroxide ions (OH⁻) may hinder Ba^2+^ binding. If the pH of a sample under measurement falls outside the sensor's operational range (2.6–6.9), it can be adjusted using acid or base to bring it within the optimal range, thereby ensuring reliable sensor performance.

### Response time, reversibility and stability of Ba^2+^ sensor

**Fig. 8:**
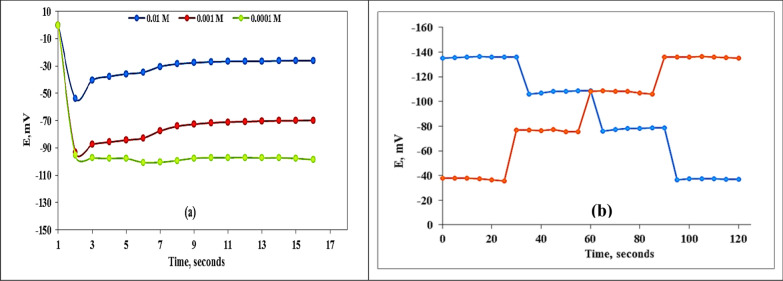
**a** Response time of Ba^2+^-sensor (type-I) for 10^–4^, 10^–3^ and 10^–2^ M, and **b** the response reversibility

Response time was estimated according to the IUPAC definition [28, 38]. It is the time between the instant when the electrode was brought into contact with the sample solution and the first instant when the emf/time slope becomes equal to the limiting value selected on the basis of experimental conditions. The response time of Ba-sensor type-I was found by recording potential-time for different Ba^2+^ concentrations. Figure 8a showed that the response time was 9 s for the barium concentrations (1.0 × 10^–2^–1.0 × 10^–4^ M) in deionized water. It is obvious that for the 10^–2^ M solution, the steady state value was − 26.6 mV, and its ± 1 mV value was − 27.6 mV, corresponding to 9 s. For 10^–3^ M solution, the steady state value was − 71.05 and its ± 1 mV value was -72.6 mV corresponding to 9 Seconds. For the 10^–4^ M solution, the steady-state value was − 97.3, and its ± 1 mV value was -97.5, corresponding to 9 s. In addition, the response time of the sensor was studied in milk, urine, juice, and tap water to assure the real applicability of the proposed sensor in the real samples. It was found that the response time slightly influenced the results to be 12 s for milk and urine and 11 s for juice and tap water. This slight variation may be attributed to the effect of the complex matrix components. The potential time changes were measured till 10 min to assure the stability of the mV readings. Statistically, all measurements were done 4 times, and the averages were taken.

The reversibility of the employed sensor was investigated by recording the potential in the Ba^2+^ ion concentrations sequence from high to low (10^–5^ to 10^–2^ M). Figure 8b's findings demonstrated that the possible reaction was reversible, which enhanced the applicability of the proposed barium-selective electrode.

In terms of stability, barium electrode stability was monitored periodically by recalibrating the sensor and evaluating its performance parameters. Over a period of ten weeks, the sensor maintained consistent Nernstian behaviour with a slope of 33 ± 0.31 mV per decade of activity, covering a dynamic working range from 10^–5^ to 10^–2^ M and achieving a detection limit of 10^–5^ M. The consistent electrode performance over this long period indicated the stability of DNA and all the membrane components. After ten weeks, the sensor suffered a slight alteration in its performance characteristics, which may be attributed to the leaching out of DOP or other electrode components.

### Selectivity of Ba^2+^ sensor

The selectivity of barium sensor type-I was determined by SSM [28]. Table 2 showed that the potentiometric selectivity coefficients exhibited the preference of the DNA-based sensor for barium ions. The average of selectivity coefficients for trivalent cations was excellent for Ba^2+^ with an order of 10^–6^, while the values of selectivity coefficients for divalent cations were lower than those for trivalent cations, which were in the order of (10^–4^, 10^–3^ and 10^–2^ M). Selectivity of several organic compounds (glycine, lactose, glucose, sucrose, arginine, and cysteine), which might be present in some tested products, was determined. The selectivity coefficient values for the mentioned compounds were less than 3.7 × 10^–4^. Furthermore, the selectivity coefficients of some common ions in milk and urine samples were studied to confirm the ability of the sensor to assess Ba^2+^ in the complex matrices and added to Table 2. The obtained selectivity coefficient values for some cations differed little due to interference from other ions and molecules present in the complex matrices of the samples. These values mean that the determination of barium in the presence of these compounds was practically available. Table 2 showed the average values of selectivity coefficients for the tested cations. The selectivity of DNA toward Ba^2+^ ions could be attributed to the ionic properties of the divalent cation [39], where Ba^2+^ has a large ionic radius (1.35 Å) and relatively lower charge density compared to other cations. The lower charge density resulted in weaker hydration, making barium ions more susceptible to the interaction with DNA’s phosphate groups. In contrast, the other cations were more strongly hydrated as they have smaller ionic radii and higher charge density, which reduced their tendency to interact with the phosphate groups. In addition, the reaction of Ba^2+^ with DNA’s phosphate groups is thermodynamically favourable due to the low solubility product of Ba-phosphate (6 × 10^–39^). Table 2 showed the obtained selectivity coefficients, which confirm that the sensor exhibits a much higher preference for Ba^2^⁺ ions compared to other divalent cations [35, 36].

**Table 2:** Values of selectivity coefficient of Barium sensor based on DNA type- I

Interference	K^Pot^_Ba_^2+^_, j_^z+^ (deionized water)	K^Pot^_Ba_^2+^_, j_^z+^ (milk)	K^Pot^_Ba_^2+^_, j_^z+^ (urine)
Cu^2+^	3.8 × 10^–2^	5.4 × 10^–2^	5.6 × 10^–2^
Ca^2+^	3.8 × 10^–2^	4.5 × 10^–2^	4.3 × 10^–2^
Hg^2+^	7.9 × 10^–2^	–	–
Mn^2+^	1.4 × 10^–2^	–	–
Mg^2+^	7.9 × 10^–2^	8.3 × 10^–2^	7.2 × 10^–2^
Zn^2+^	2.2 × 10^–3^	3.5 × 10^–3^	3.7 × 10^–3^
Pb^2+^	1.3 × 10^–4^	–	–
Ni^2+^	5.4 × 10^–4^	5.9 × 10^–4^	5.6 × 10^–3^
Cr^3+^	5.1 × 10^–3^	5.6 × 10^–3^	5.4 × 10^–3^
Sr^2+^	2.8 × 10^–3^	3.2 × 10^–3^	4.1 × 10^–3^
Na^+^	7.6 × 10^–5^	8.1 × 10^–5^	9.5 × 10^–5^
K^+^	9.8 × 10^–5^	1.5 × 10^–4^	3.4 × 10^–4^
Fe^3+^	3.06 × 10^–6^	4.2 × 10^–6^	5.5 × 10^–6^
Al^3+^	3.06 × 10^–6^	–	–
Fe^2+^	3.2 × 10^–3^	4.1 × 10^–3^	3.6 × 10^–3^
Sn^2+^	5.6 × 10^–4^	–	–
Ag^+^	4.3 × 10^–4^	–	–
Glycine	4.5 × 10^–6^	–	–
Lactose	5.2 × 10^–7^	–	–
Glucose	1.3 × 10^–6^	–	–
Sucrose	1.4 × 10^–6^	–	–
Arginine	3.7 × 10^–4^	–	–
Cysteine	8.5 × 10^–5^	–	–

### Determination of barium in its samples

Different barium-spiked samples (milk, juice, urine, and tap water) were analyzed by using the proposed barium sensor. The tested samples were measured four times (n = 4), and the sensor was soaked for two hours in the sample before the measurement to obtain accurate results. The obtained results showed that the sensor was successfully used for measuring barium in different samples. The obtained recovery values for milk, juice, tap water, and urine were 97.57–98.30, 96.35–97.67, 96.07–97.45, and 96.60–98.83%, respectively. The assessed concentrations were selected higher or equal to the detection limit of the sensor (10^–5^ M). The results were shown in Table 3.

**Table 3:** Results of determination of barium in spiked samples including the inter-day and the intra-day results of analysis

Samples	mg/L-Taken	mg/L-found	%Recovery	Inter-day Recovery%	Intra-day-Recovery%
Milk	2.06	2.01	97.57	97.33	98.10
	2.75	2.72	98.90	97.91	98.55
	4.12	4.05	98.30	98.75	99.70
	5.49	5.43	98.90	97.57	98.60
	6.87	6.79	98.84	98.22	98.28
Juice	1.37	1.32	96.35	94.54	95.30
	2.75	2.68	97.45	96.20	97.05
	4.12	3.99	96.84	97.14	97.44
	5.49	5.35	97.5	95.13	96.75
	6.87	6.71	97.67	95.78	96.21
Tap water	1.37	1.31	96.35	95.59	96.39
	2.75	2.68	97.45	96.16	96.37
	4.12	3.99	96.90	97.28	97.83
	5.49	5.34	97.30	96.70	98.64
	6.87	6.60	96.07	97.88	98.22
Urine	2.55	2.46	96.60	96.25	97.40
	3.22	3.13	97.20	96.74	97.54
	4.52	4.45	98.45	97.40	98.13
	5.12	5.06	98.83	97.92	98.85

n = 4-determinations

Reproducibility was the variation in readings when a different person measures the same part (or quantity) many times, using the same equipment and the same method, under the same conditions. The measurements were done for several spiked samples with different added concentrations of Ba^2+^. In addition, the inter-day and the intra-day results were included for assuring the reproducibility. The obtained results of the inter-day and intra-day were not outside the accepted limits. The results were shown in Table 3.

### Comparison of the sensor characteristics with previous sensors

DNA-based sensors offer notable benefits, including rapid response, high selectivity, excellent stability, and versatility. They are also cost-effective and utilize an eco-friendly, renewable natural material, making them highly suitable for accurate and reliable detection of metal ions across several applications under diverse conditions. Additionally, Table 4 presents a comparative analysis of the performance characteristics of the proposed sensor against previously reported ones, highlighting its advantages. The obtained results showed that the use of DNA as a polyionic ionophore for barium gave a super-Nernstian slope and has the fastest response time compared to the previous Ba^2+^ ionophores. In addition, the doping technique was used for preparing the sensor, which is an easy method for sensor fabrication [18–21]. The proposed sensor showed super-Nernstian slope 33.15 mV/decade, which means that the sensor will be more sensitive for small changes in concentrations. In contrast, the previously reported sensors were based on hazard organic ionophores that require several steps to be prepared in addition to high degree of precautions and extreme conditions should be maintained. Furthermore, some sensors suffered from interferences and sub-Nernstian slope that made them less sensitive to small changes in concentrations [40–48].

**Table 4:** Comparison between of the performance characteristics of the proposed barium DNA- based sensor with the previous Ba-sensors

Ionophore	Slope mV/Decade^−1^	Linear range, M	Response Time, s	DL	pH	References
Dimethyl 1-Acetyl-8-oxo-2,8-dihydro- 1H-pyra-zolo[5,1-a]isoindole-2,3-dicarboxylate	29.7	1.0–10^−6^–1.0 × 10^–1^	10	7.6 × 10^–7^	3.0–11.0	[18]
4ʹ,4ʺ(5ʺ)-Di-tert-butyldibenzo-18-Crown-6	25.23	1.0 × 10^–7^–1.0 × 10^–1^	18	8.9 × 10^–8^	2.0–12.4	[19]
Complex ion associate barium (II)–Rose Bengal	28.5	5.0 × 10^–5^–10^–1^	20	2.5 × 10^–6^	4.5–10.0	[20]
3-deoxy-d-erythro-hexos-2-ulose bis (thiosemicarbazone)	29.6	1.0 × 10^–6^–1.0 × 10^–2^	15	5.6 × 10^–7^	2.6–11.0	[21]
2-(2-formylphenoxy) acetic acid	28.9	1.0 × 10^–6^–1.0 × 10^–1^	< 10	5.54 × 10^–7^	6.0–9.0	[40]
dibenzo-24-crown-8	30.1	1.0 × 10^–6^–1.0 × 10^–3^	< 10	6.1 × 10^–7^	4.1–9.0	[41]
benzo-15-crown-5	29.1	1.0 × 10^–6^–1.0 × 10^–1^	20	0.6 × 10^–5^	2.0–6.0	[42]
Calix[4]crown-6	29.10	1.0 × 10^–6^–1.0 × 10^–3^	10	7.1 × 10^–7^	4.0–10.0	[43]
a macrocyclic tetrabasic ligand	29.70	3.6 × 10^–6^–1.0 × 10–1	9	1.9 × 10^–6^	2.5–7.5	[44]
4–4′-Methylenediantipyrine	29.7	1.0 × 10^–6^–1.0 × 10^–2^	15	5.2 × 10^–7^	3.4–10.6	[45]
2,3,4-pyridine-1,3,5,7,12-pentaazacyclopentadeca-3-ene	30.0	1.41 × 10^–6^–1.0 × 10^–1^	18	–	2.5–7.0	[46]
DDB liver drug	30.0	1.0 × 10^–5^–1.0 × 10^–1^	–	5.0 × 10^–6^	4.0–9.0	[47]
neutral bidentate organophosphorus compounds	30.0	1.0 × 10^–5^–1.0 × 10^–1^	60	5.0 × 10^–6^	3.0–11.0	[48]
DNA	33.15	1.0 × 10^–5^–1.0 × 10^–2^	9	1 × 10^–5^	2.6–7.0	Present work

## Materials and methods

### Reagents

All of the used reagents in this work were of high purity. Deoxyribonucleic acid (DNA) (TiTAN BIOTECH LTD, Rajasthan, India) was used as an ionophore. Potassium tetrakis(4-chlorophenyl) borate (T*p*ClPB) of formula (ClC_6_H_4_)_4_BK was purchased from (Sigma/Aldrich, Taufkirchen, Germany). The used plasticizers were either dioctyl phthalate (DOP) (C_24_H_38_O_4_) (Aldrich, Taufkirchen, Germany) or diethyl-phthalate (DEP) (C_12_H_14_O_4_) (Aldrich, Taufkirchen, Germany). Tetrahydrofuran (THF) was used for dissolving the membrane components. Poly vinyl chloride (PVC) was used as membrane matrix was purchased from (Shintech, Texas, U.S.A). Barium chloride dihydrate(BaCl_2_.2H_2_O) was purchased from (Adwic Chemical Company, Cairo, Egypt). The sulphates, chlorides or nitrates of selected cations K^+^, Na^+^, Ag^+^, NH_4_^+^, Cu^2+^, Ni^2+^, Mg^2+^, Sr^2+^, Zn^2+^, Hg^2+^, Mn^2+^, Fe^2+^, Sn^2+^, Fe^3+^, Al^3+^, Cr^3+^,,and Pb^2+^ (Adwic Chemical Company, Cairo, Egypt) were used to detect the sensor selectivity. Glycine, lactose, sucrose, arginine and cysteine were purchased from (Adwic Chemical Company, Cairo, Egypt).

### Characterization of the membrane composition

FT-IR characterization, the functional groups analysis of the PUFB was characterized by using infrared spectrometer (Alpha-1 00523), (Nucleic Acids Research Center, Faculty of Science, Zagazig University, Zagazig, Egypt). EDX analysis and SEM-photos were done by scanning electron microscopy (odel ZEISS-EVO 15–UK (National Research Centre, Cairo, Egypt). Samples were gold sputtered for 5 min and examined by a scanning electron microscope operated at an accelerating voltage of 25 kV.

### Electrode preparation

The preparation of the membrane was made by mixing 60 mg of PVC, 120 mg of plasticizers (DEP or DOP), and 2 mg of tetra phenyl borate (TPB). Then, mixed with either 2, 4, or 6 mg of DNA as an ionophore. After that, these materials were dissolved in about 5 ml of THF, while the DNA was dispersed into the solvent. The components were mixed well and left a little till (THF) was evaporated and a paste was obtained. A clean silver wire was immersed in the prepared cocktail for about 30 s many times until a coat was formed. After that, the silver wire was left at room temperature until it dried. Then, the formed sensor was soaked for 24 h at room temperature in a 1 × 10^−2^ M solution of barium chloride_._

### Potentiometric measurements

A range of barium solutions (10^−8^ to 10^−2^ M) were created for emf measurements. 25 mL aliquots of the produced Ba^2+^solutions were placed in a 25 mL beaker. The suggested barium electrode and Ag/AgCl (Jenway, model 924017) reference electrode were immersed in each concentration. The exhibited potential was recorded for each tested concentration. A calibration graph was created based on the relationship between the measured potential and (−log [Ba^2+^]). The utilised cell may be stated as follows:

$${\text{Ag }}/{\text{ AgCl Reference }}//{\text{ Test solution }}/{\text{ PVC Membrane }}/{\text{ Ag}}$$

### Composition and pH influence

The membrane composition was optimized by varying the ratios of DNA. The suggested electrodes used 2, 4, or 6 mg of the ionophore comprising DOP or DEP as plasticizers. All electrodes were calibrated using barium solutions (10^−8^ to 10^−2^ M), and calibration graphs were built to discover the best performance characteristics.

For studying the pH effect, the reference electrode, the proposed sensor, and the glass electrode were immersed in 10^–3^ M barium solution to be measured. Either 0.1 M sodium hydroxide or 0.1 M hydrochloric acid were used to adjust the pH of the solution in the range of 2 to 10. Plotting of the potential values versus the measured pH values was done.

### Response time and reversibility

The Ba^2+^-sensor was dipped into barium solutions (10^−5^, 10^−4^, 10^−3^, and 10^−2^ M) to measure the period needed to establish a steady potential reading (± 1 mV) following the change of Ba^2+^ concentration. The response time was determined by recording the measured potential and graphing it against time. For reversibility, the potential in the succession of high to low Ba^2+^ ion concentrations (10^–5^ to 10^–2^ M) was measured.

### Selectivity studies

Three concentrations (10^–4^, 10^–3^ and 10^–2^ M) of various cations were used for the measurements to calculate selectivity coefficient values in deionized water, milk and urine samples. They were measured by the IUPAC separate solution method [28] by using the following Eq. (3):

3 $${\text{Log K}}^{{{\text{pot}}}} _{{{Ba}} ^{{2 + }}} ,_{{{\text{J}}}} ^{{{{\text{z}} + }}} = \, \left[ {\left( {{\text{E}}_{{\text{j}}} {-}{\text{ E}}_{{{{\text{Ba}}}}^{{{2} + }}} } \right)/{\text{S}}} \right] + \, \left[ {\left( {{1} - \left( {{\text{Z}}_{{{{\text{Ba}}}}^{{{2} + }}} /{\text{Z}}_{{\text{j}}} } \right)} \right){\text{ log }}\left( {{\text{a}}_{{{{\text{Ba}}}}^{{{2} + }}} } \right)} \right]$$

where, E_i_ is the potential of barium solution. E_j_: is the potential measured in 10^–2^, 10^–3^, and 10^–4^ M solutions of the interfering cations; ∆E: equals (E_j_–E_*Ba*_^*2*+^); S: is the slope of the sensor calibration plot; and *Z*_*Ba*_^*2*+^*/Z*_*j*_: is the valance of Ba^2+^ and the interfering ion, respectively.

### Determination of Ba^2+^ in spiked samples

Several samples (biological, food and tap water) were spiked with barium ions to study the ability of the sensor to measure Ba^2+^ in the presence of different cations. Milk and juice were sold from local market, and tap water was obtained from laboratory of analytical chemistry (Zagazig, Egypt). Urine sample was collected from healthy volunteer. 25-mL aliquots of Ba^2+^ samples were transferred into beakers. Then, both the sensor and the reference electrode were dipped into each solution. The potential value of each sample was measured. All of the obtained mV-values of each sample were referred to a previously prepared calibration graph to find the Ba^2+^ concentrations in the samples.

## Conclusion

From the obtained results, it can be concluded that DNA as a polyionic ionophore incorporated into the PVC matrix can be successfully applied for the construction of a Ba^2+^-coated wire selective electrode. The suggested sensor improved the response time of the Ba^2+^-selective electrode compared to previously reported electrodes. In addition, the constructed sensor demonstrated strong selectivity for Ba^2+^ ions, application across wide pH ranges, and high sensitivity. Depending on the obtained optimum performance characteristics of the sensor, the estimation of Ba^2+^ ions in milk, juice, urine and tap water was successfully achieved with high recovery.

## Data Availability

The data that support the findings of this study are available from the corresponding author upon reasonable request.

## Abbreviations

DNA: Deoxyribonucleic acid
SEM: Scanning electron microscopy
EDX: Energy dispersive x-ray
PVC: Polyvinyl chloride
THF: Tetrahydrofuran
SSM: Separate-solution method
M: Molar
ISEs: Ion-selective electrodes
DOP: Dioctyl phthalate
DEP: Diethyl phthalate
